# Molecular Detection of Feline Hemotropic *Mycoplasma* from Blood Samples of Sick Cats: A Comparative Study Between a Commercial Nucleic Acid Test Card and Conventional Polymerase Chain Reaction

**DOI:** 10.3390/vetsci13090978

**Published:** 2026-09-17

**Authors:** Burin Nimsuphan, Sakchai Ruenphet, Chanya Kengradomkij, Nutsuda Klinkaew, Ketsarin Kamyingkird

**Affiliations:** 1 Department of Parasitology, Faculty of Veterinary Medicine, Kasetsart University, Lad Yao, Chatuchak, Bangkok 10900, Thailand; fvetbrn@ku.ac.th (B.N.); fvetcyk@ku.ac.th (C.K.); fvetnuk@ku.ac.th (N.K.); ketsarin.ka@ku.th (K.K.); 2 Animal Biotechnology Program, Faculty of Veterinary Medicine, Mahanakorn University of Technology, 140 Cheum-Sampan Rd., Nong Chock, Bangkok 10530, Thailand

**Keywords:** *Candidatus* Mycoplasma haemominutum, *Candidatus* Mycoplasma turicensis, conventional PCR, *Mycoplasma haemofelis*, nucleic acid test card, 16S rRNA gene

## Abstract

Feline hemotropic mycoplasmosis presents a diagnostic challenge in clinical practice because of the variable pathogenicity of *Mycoplasma* species and the limited sensitivity of conventional blood smear examination, highlighting the need for rapid and accurate point-of-care molecular diagnosis. This study aimed to evaluate the diagnostic performance of the Pluslife^®^ Feline Hemotropic *Mycoplasma* (FHM) nucleic acid test card and to determine the detection frequency of the pathogen among clinically ill cats presented to a veterinary teaching hospital in Bangkok, Thailand. Whole blood samples were collected from 114 clinically ill cats presented to Kasetsart University Veterinary Teaching Hospital between September 2025 and May 2026. The nucleic acid test card assay was evaluated against conventional PCR targeting the 16S rRNA gene as the reference method. PCR identified ‘*Candidatus* Mycoplasma haemominutum’ (*C*Mhm) as the predominant *Mycoplasma* species (45.61%), followed by *Mycoplasma haemofelis* (*Mhf*; 5.26%) and ‘*Candidatus* Mycoplasma turicensis’ (*C*Mt; 0.88%). Compared with conventional PCR, the nucleic acid test card assay demonstrated a sensitivity of 93.22%, specificity of 96.36%, overall accuracy of 94.74%, and precision of 96.49%, with results available within 30 min. Sequence analysis of discordant samples confirmed *C*Mhm in four false-negative specimens, suggesting that low bacterial burdens or other assay-related factors may have contributed to the discordant results. Overall, the Pluslife^®^ FHM assay demonstrated high diagnostic performance and may provide a practical rapid molecular point-of-care tool to support timely diagnosis and clinical decision-making in cats with suspected hemotropic mycoplasmosis.

## Simple Summary

Feline hemotropic mycoplasmosis is a clinically important infectious disease of domestic cats that can cause severe, potentially life-threatening hemolytic anemia, systemic complications, and persistent carrier states. Because clinical signs are often nonspecific and conventional blood smear examination has limited sensitivity, particularly in subclinical or low-burden infections, rapid and reliable point-of-care diagnostic methods are needed to support timely diagnosis and appropriate clinical management. Although PCR provides high diagnostic sensitivity, its dependence on specialized laboratory infrastructure can limit accessibility and prolong turnaround times for many primary-care veterinary clinics. To address these limitations, we evaluated the Feline Hemotropic *Mycoplasma* (FHM) nucleic acid test card, a rapid point-of-care molecular assay which provided the results within approximately 30 min. Blood samples collected from clinically ill cats presented to a veterinary teaching hospital in Bangkok, Thailand, were tested using both the Pluslife^®^ FHM test card assay and conventional PCR as the reference method. Compared with conventional PCR, the Pluslife^®^ FHM assay demonstrated high diagnostic performance, with high sensitivity and specificity.

## 1. Introduction

Feline hemotropic mycoplasmosis, commonly referred to as feline hemoplasmosis, is a significant transboundary vector-borne infectious disease whose signs range from mild or silent carrier stages to life-threatening hemolytic anemia and systemic illness in domestic and wild felines worldwide [1,2,3]. The disease is primarily caused by three hemotropic, cell wall-deficient, pleomorphic bacterial species within the class Mollicutes that obligately parasitize the epicellular surfaces of host erythrocytes: *Mycoplasma haemofelis* (*Mhf*), ‘*Candidatus* Mycoplasma haemominutum’ (*C*Mhm), and ‘*Candidatus* Mycoplasma turicensis’ (*C*Mt) [4,5,6]. Among these three species, *Mhf* is the most virulent and frequently causes severe hemolytic anemia accompanied by extensive intravascular and/or extravascular hemolysis [7]. In contrast, *C*Mhm and *C*Mt have traditionally been regarded as less virulent species, typically causing subclinical infections or only mild hematological abnormalities in immunocompetent cats [4,5,8]. Nevertheless, recent reports have documented cases of severe acute anemia associated with *C*Mhm as the sole detected pathogen, indicating that this species may occasionally cause severe disease. Furthermore, mixed hemotropic *Mycoplasma* infections and concurrent retroviral infections can substantially worsen clinical manifestations and increase disease severity [4,5,8,9].

The global distribution and epidemiological dynamics of feline hemotropic mycoplasmas are highly heterogeneous and vary considerably across geographic regions. A recent systematic review and meta-analysis estimated a global pooled hemotropic *Mycoplasma* prevalence of 19.8% in domestic cats, with *C*Mhm identified as the predominant species, followed by *Mhf* and *C*Mt [10]. Transmission is believed to occur primarily through hematophagous arthropods, particularly the cat flea (*Ctenocephalides felis*) and the brown dog tick (*Rhipicephalus sanguineus*), which are considered important potential vectors [4,5,6,11,12]. In addition to vector-borne transmission, horizontal spread through aggressive interactions, especially bite wounds, has been widely documented. Consequently, intact outdoor male cats and cats living in high-density shelter environments consistently exhibit higher infection rates [4,6,13]. More recently, molecular surveillance studies have revealed the presence of atypical hemotropic *Mycoplasma* species, including *Mycoplasma wenyonii*, the primary causative agent of bovine hemotrophic mycoplasmosis, in companion animals and feline hosts. These findings suggest possible cross-species transmission or previously unrecognized host associations [4,6]. Collectively, these observations underscore the importance of comprehensive molecular surveillance within a One Health framework to improve understanding of host adaptation, pathogen transmission, and the emergence of novel infection risks [6].

In Southeast Asia, particularly in Thailand, feline hemoplasmosis is an endemic infectious disease affecting diverse feline populations [5,8,14]. Molecular surveillance studies have reported prevalence rates ranging from 11.2% in hospitalized cats to 38.05% in semi-domesticated and feral cats inhabiting public areas and temple environments [6,8,15]. Among reported Thai isolates, *C*Mhm is the predominant species, whereas *Mhf* occurs less frequently and *C*Mt is detected only sporadically [14]. Given the high prevalence of infection and the predominance of *C*Mhm in Thailand, accurate and timely diagnosis remains essential for effective disease management. Despite its clinical and veterinary importance, early diagnosis of feline hemoplasmosis faces substantial technical challenges [4,5]. Light microscopic examination of Giemsa-stained blood smears remains widely used in resource-limited veterinary settings. However, this method has limited sensitivity (approximately 51.47%), may fail to detect low-level bacteremia, and is susceptible to observer bias and staining artifacts [4,5,16]. Molecular diagnostic methods, particularly conventional PCR targeting the 16S rRNA gene and multi-locus sequence analysis (MLSA), are widely regarded as reference standards because of their high analytical sensitivity and specificity [4,5,8,17,18]. Nevertheless, conventional PCR-based methods require specialized laboratory infrastructure, relatively high operation costs, and longer turnaround times, limiting their applicability for rapid field-based screening and outbreak response.

To overcome the limitations of both centralized PCR assays and conventional microscopic screening, several advanced point-of-care testing (POCT) technologies have been developed to provide rapid and reliable molecular diagnosis. Among these technologies, recent validation studies conducted in companion animal and livestock settings have demonstrated the promising diagnostic performance of RNase hybridization-assisted amplification (RHAM) technology. RHAM is an isothermal molecular assay that integrates loop-mediated isothermal amplification (LAMP) with an RNase HII-mediated detection system within a fully automated closed-cartridge platform. This design provides qPCR-comparable analytical sensitivity and specificity, generates results in less than 35 min, and minimizes the risk of aerosol-mediated cross-contamination [19,20,21]. Although RHAM has been successfully validated for detecting other blood-borne pathogens and infectious retroviruses in companion animals, evidence supporting its diagnostic performance for feline hemotropic mycoplasmas under clinical field conditions remains limited. Consequently, further evaluation is required to determine its suitability for routine diagnosis of feline hemoplasmosis.

Therefore, this study aimed to evaluate the diagnostic performance of the commercial Pluslife^®^ Feline Hemotropic *Mycoplasma* (FHM) nucleic acid test card, including its sensitivity, specificity, and precision, by direct comparison with conventional PCR targeting the 16S rRNA gene. Using blood samples collected from cats presented to a veterinary teaching hospital in Bangkok, Thailand, the study also assessed the clinical utility of this rapid point-of-care molecular assay for supporting timely diagnosis and clinical decision-making in feline hemoplasmosis. The study further aimed to characterize the detection frequency and molecular epidemiology of feline hemotropic mycoplasmas in Thailand.

## 2. Materials and Methods

### 2.1. Ethical Approval and Informed Consent

The study protocol was approved by the Animal Research Ethics Committee, Faculty of Veterinary Medicine, Mahanakorn University of Technology, Thailand (approval no. ACUC-MUT-2025/011), and the Institutional Animal Care and Use Committee of Kasetsart University, Thailand (approval no. ACKU66-VET-002), before sample collection commenced in September 2025. Written informed consent for study participation and publication was obtained from all cat owners before enrollment.

### 2.2. Study Period and Location

Between September 2025 and May 2026, blood samples were collected from sick cats presented at the Veterinary Teaching Hospital and sent to the Diagnostic Parasitology Laboratory, Faculty of Veterinary Medicine, Kasetsart University, Bangkok, Thailand, for subsequent analysis.

### 2.3. Sample Collection and Study Protocol Design, and Sample Size Justification

A total of 114 cats of various ages and breeds were enrolled in the study. All enrolled cats exhibited one or more clinical signs potentially compatible with hemotropic *Mycoplasma* infection, including fever, pale mucous membranes, anemia, jaundice, and weight loss. The minimum required sample size was estimated using Buderer’s formula for diagnostic accuracy studies. Assuming an anticipated sensitivity and specificity of 90%, a 95% confidence level, and an expected hemoplasma prevalence of approximately 45% among clinically ill cats in Thailand based on historical surveillance, the minimum required sample size was calculated to be 107 cats. The final enrollment of 114 cats therefore exceeded the minimum required sample size and was considered adequate for evaluating the diagnostic performance and agreement of the RHAM assay relative to the reference method.

Whole blood samples were collected into EDTA tubes and divided into two aliquots upon receipt to ensure that both diagnostic assays were performed using material derived from the same specimen. To minimize potential identification and observer bias, each specimen was assigned an anonymized tracking code by an independent technician who was not involved in performing either diagnostic assay. The first aliquot was tested immediately using the Pluslife^®^ FHM nucleic acid test card by an operator blinded to the conventional PCR results and the cat’s detailed medical records. The second aliquot was stored at −20 °C until batched DNA extraction and conventional PCR analysis by independent laboratory personnel who were blinded to the Pluslife^®^ assay results. Results from the two diagnostic methods were unblinded and tabulated only after all assays had been completed. Because of the routine diagnostic submission workflow of the parasitology diagnostic unit, detailed hematological data, including complete blood count, packed cell volume values, and epidemiological and lifestyle information, such as ectoparasite prevention history and indoor/outdoor status, were not systematically available for all enrolled cats.

### 2.4. RNase Hybridization-Assisted Amplification (RHAM) Assay

The RHAM assay for detecting *C*Mhm, *Mhf*, and *C*Mt comprises three main components: the Pluslife Integrated Nucleic Acid Testing Device, a nucleic acid releaser tube, and the Pluslife^®^ Feline Hemotropic *Mycoplasma* (FHM) nucleic acid test card (Guangzhou Pluslife Biotech, Guangzhou, China). All testing procedures were performed according to the manufacturer’s instructions.

Before testing, the device was preheated and connected to the Pluslife Pet application (version 1.7) on a computer. After preheating, 50 µL of whole blood was transferred from the EDTA specimen to the nucleic acid releaser tube using a disposable Pasteur pipette. The tube was then incubated at 65 °C for 5 min to facilitate nucleic acid release. The resulting lysate was transferred to the designated sample inlet of the FHM nucleic acid test card. The test card was maintained in an upright position for 15 s, after which the air button on the cartridge cap was pressed. The card was then shaken briefly and inserted into the Integrated Nucleic Acid Testing Device. The assay was initiated by selecting the “Start” function in the Pluslife Pet application, and test results were displayed after approximately 30 min.

### 2.5. Conventional Polymerase Chain Reaction (PCR)

Genomic DNA was extracted from EDTA-anticoagulated whole blood using the NucleoSpin^®^ Blood kit (Macherey-Nagel GmbH & Co., Düren, Germany) according to the manufacturer’s instructions. DNA was eluted in a final volume of 100 µL and stored at −20 °C until subsequent PCR analysis.

Hemotropic *Mycoplasma* DNA was detected by conventional PCR targeting the 16S rRNA gene. The primer pair described by Criado-Fornelio et al. [22] was used: forward primer 5′-ATACGGCCCATATTCCTACG-3′ and reverse primer 5′-TGCTCCACCACTTGTTCA-3′. Each PCR was performed in a final volume of 20 µL containing 1× PCR buffer, 1.5 mM MgCl_2_, 0.2 mM dNTPs, 0.1 µM of each primer, 1.0 U of Taq DNA polymerase (Invitrogen™, Thermo Fisher Scientific, Carlsbad, CA, USA), and 2 µL of DNA template.

To monitor assay performance and potential contamination, positive and no-template controls were included in each PCR run. Sequence-confirmed *Candidatus* Mycoplasma haemominutum DNA served as the positive control, whereas nuclease-free water served as the no-template control. PCR products were analyzed by agarose gel electrophoresis as previously described [23,24,25,26,27]. The expected amplicon size was approximately 580 to 620 bp, depending on the hemoplasma species.

### 2.6. Analysis

The detection frequency of hemotropic *Mycoplasma* infection was calculated as the proportion of PCR-positive cats among all cats tested and expressed as a percentage. The diagnostic performance of the Pluslife^®^ FHM nucleic acid test card was evaluated using conventional PCR as the reference method. Pluslife^®^ FHM assay results were classified as true positives, true negatives, false positives, or false negatives according to their agreement with the conventional PCR results. Sensitivity, specificity, accuracy, and precision (positive predictive value) of the Pluslife^®^ FHM assay relative to conventional PCR were calculated using the following standard formulas [28,29]:

$$Diagnostic\;sensitivity=\frac{No.\;of\;true\;positive}{No.\;of\;true\;positive+No.\;of\;false\;negative}\times 100$$

$$Specificity=\frac{No.\;of\;true\;negative}{No.\;of\;true\;negative+No.\;of\;false\;positive}\times 100$$

$$Accuracy=\frac{\left(No.\;of\;true\;positive\right)+(No.\;of\;true\;negative)}{\left(No.\;of\;true\;positive\right)+\left(No.\;of\;true\;negative\right)+\left(No.\;of\;false\;positive\right)+(No.\;of\;false\;negative)}\times 100$$

$$Precision=\frac{No.\;of\;true\;positive}{No.\;of\;true\;positive+No.\;of\;false\;positive}\times 100$$

### 2.7. DNA Sequencing

To investigate the causes of discordant diagnostic results and confirm hemotropic *Mycoplasma* species, all amplicons of PCR-positive samples, including four discordant samples (negative by the Pluslife^®^ FHM nucleic acid test card but positive by conventional PCR) were subjected to DNA sequencing. After 16S rRNA gene conventional PCR, the positive products were cut from the gel and purified using a PCR clean-up gel extraction kit (NucleoSpin^®^ Gel and PCR Clean-up, MACHEREY-NAGEL GmbH & Co, Düren, Germany). Subsequently, the purified product was submitted for Sanger DNA sequencing (Macrogen, Beotkkot-ro, Geumcheon-gu, Seoul, Republic of Korea). For molecular identification, the consensus partial 16S rRNA sequences were compared with reference sequences in the National Center for Biotechnology Information (NCBI) database using the Basic Local Alignment Search Tool (BLAST). Species identification was based primarily on the highest percentage sequence identity and corresponding E-values, while the closest matching reference accession numbers were recorded. The sequencing analysis included four false-negative samples (66, 68, 105, and 107) to determine whether they represented recognized feline hemotropic *Mycoplasma* species, including *C*Mhm, *Mhf*, and *C*Mt, or other non-target or cross-species.

## 3. Results

### 3.1. Demographic Characteristics of the Study Population

The demographic characteristics of the study population, comprising 114 cats, are summarized in Table 1. Male cats were the majority of the population (*n* = 76, 66.67%), whereas female cats accounted for the remaining 38 animals (33.33%). The largest age group comprised cats aged 1–5 years (*n* = 47, 41.23%), followed by those aged 6–10 years (*n* = 41, 35.96%), cats older than 11 years (*n* = 17, 14.91%), and kittens younger than 1 year (*n* = 9, 7.89%). Domestic shorthair (DSH) cats were the predominant breed, accounting for 87 animals (76.32%). The remaining cats were purebred; Scottish Fold (*n* = 15, 13.16%) was the majority; the other purebreds are shown in Table 1.

### 3.2. Diagnostic Performance of the Pluslife^®^ FHM Nucleic Acid Test Card Compared with Conventional PCR

The diagnostic performance of the Pluslife^®^ FHM nucleic acid test card was evaluated using conventional PCR as the reference standard. Individual test results for all 114 clinical specimens are provided in Supplementary Table S1, while the cross-tabulation results and calculated diagnostic performance metrics are summarized in Table 2.

Of the 114 samples analyzed, 55 were concordantly positive (true positive) and 53 were concordantly negative (true negative) by both methods. Six discordant results were identified: four false-negative samples (Nos. 66, 68, 105, and 107), which were negative by the Pluslife^®^ FHM assay but positive by conventional PCR; and two false-positive samples (Nos. 24 and 26), which were positive by the Pluslife^®^ FHM assay but negative by conventional PCR.

Specifically, samples No. 24 and 26 generated positive *C*Mhm signals with the Pluslife^®^ FHM assay but yielded no detectable amplicon bands following conventional PCR and agarose gel electrophoresis. As conventional PCR served as the reference standard, these two samples were classified as false positives for diagnostic performance evaluations. Furthermore, the absence of PCR amplicons prevented Sanger sequencing from being performed to resolve the discordant results.

Relative to conventional PCR, the Pluslife^®^ FHM assay demonstrated a sensitivity of 93.22% and a specificity of 96.36%. Overall accuracy was 94.74%, and precision (positive predictive value) was 96.49%.

### 3.3. Molecular Characterization and Sequence Analysis of Discrepant Isolates

Bidirectional Sanger sequencing followed by BLASTn analysis was performed on partial 16S rRNA gene amplicons from all PCR-positive samples (Table S1), including the four discordant (false-negative) samples, to investigate the observed diagnostic discrepancies. As shown in Table 3, BLASTn analysis identified all four false-negative samples as the target feline hemoplasma species *C*Mhm. These four isolates showed high sequence similarity to reference *C*Mhm strains, with nucleotide identities ranging from 95.38% to 99.49% and E-values of 0.0.

### 3.4. Detection Frequency and Species Distribution of Hemotropic Mycoplasma

The detection frequencies and species distributions of hemotropic *Mycoplasma* were compared between conventional PCR and the Pluslife^®^ FHM assay in 114 clinically ill cats presented to Kasetsart University Veterinary Teaching Hospital between September 2025 and May 2026 (Table 4).

Conventional PCR identified *C*Mhm as the most frequently detected single-species infection (*n* = 52/114, 45.61%), followed by *Mhf* (*n* = 6/114, 5.26%) and *C*Mt (*n* = 1/114, 0.88%). By comparison, the Pluslife^®^ FHM assay identified single-species *C*Mhm infection in 48 cats (48/114, 42.11%), *Mhf* infection in 5 cats (5/114, 4.39%), and *C*Mt infection in 1 cat (1/114, 0.88%). Notably, the Pluslife^®^ FHM assay detected co-infections of hemotropic *Mycoplasma* species (*C*Mhm and *Mhf*) in three DSH cats, whereas no co-infections were detected by conventional PCR.

## 4. Discussion

Rapid and accurate POCT platforms have become increasingly important in veterinary diagnostics, particularly for management of infectious diseases in clinical and field settings [19,20,21]. In this context, we evaluated the diagnostic performance of the Pluslife^®^ FHM nucleic acid test card relative to conventional PCR targeting the 16S rRNA gene in naturally infected cats presented to a veterinary teaching hospital in Thailand. Relative to conventional PCR, the Pluslife^®^ FHM assay demonstrated a sensitivity of 93.22%, specificity of 96.36%, overall accuracy of 94.74%, and precision (positive predictive value) of 96.49%, indicating strong diagnostic performance in the study population. These findings are broadly consistent with previous studies of isothermal nucleic acid amplification methods in veterinary diagnostics. Loop-mediated isothermal amplification (LAMP) and recombinase polymerase amplification (RPA) assays have shown favorable diagnostic performance and rapid turnaround times for several veterinary pathogens, including feline vector-borne agents, feline coronavirus, canine parvovirus, and bovine hemoplasmas [30,31,32,33,34]. More directly, RHAM-based assays developed for companion animal pathogens, including feline leukemia virus (FeLV), feline immunodeficiency virus (FIV), *Ehrlichia canis*, and several human pathogens, have demonstrated diagnostic performance approaching that of laboratory-based molecular methods, while their closed-cartridge design may reduce the risk of post-amplification carry-over contamination [19,20,21,35,36,37].

Hemotropic *Mycoplasma* infections were frequently detected among hospitalized cats in this study, with *C*Mhm representing the predominant species detected by both conventional PCR (45.61%) and the Pluslife^®^ FHM assay (42.11%). The predominance of CMhm observed in the present study is consistent with recent global meta-analysis that identified CMhm as the most frequently detected feline hemoplasma species worldwide [10]. Our findings are also consistent with previous molecular epidemiological studies conducted in Thailand. For example, Kamyingkird et al. [15] reported *C*Mhm as the predominant hemotropic *Mycoplasma* species among stray cats living in Bangkok monasteries, with an individual prevalence of 24.53% and detection at all hemoplasma-positive sampling sites. Similarly, Bui et al. [8] confirmed through multi-locus sequence analysis that *C*Mhm remained the most frequently detected hemotropic *Mycoplasma* species among domestic cats in Thailand. Most infected cats in the present study were male and DSH cats, reflecting the demographic composition of the study population. Previous studies have suggested that behavioral factors, including territorial fighting and outdoor roaming, increase exposure to ectoparasites such as *Ctenocephalides felis*, thereby facilitating the transmission of hemotropic *Mycoplasma* species [10,15].

Investigation of discordant results between the Pluslife^®^ FHM assay and conventional PCR provided important insights into potential sources of diagnostic disagreement. Sequence analysis of the four conventional PCR-positive/Pluslife-negative samples provided additional information on the identity and genetic characteristics of the detected organisms. BLASTn analysis showed that sequences obtained from all four discordant samples (Nos. 66, 68, 105 and 107) most closely matched *C*Mhm reference sequences, with sequence identities ranging from 95.38% to 99.49%. One possible explanation for these false-negative results is a bacterial burden below the analytical detection limit of the Pluslife^®^ FHM assay. Although bacterial loads were not quantified in the present study, reduced detection at low target concentrations has been reported for RHAM-based assays developed for other animal pathogens [19,20,21]. Similar limitations at low target concentrations have been reported for other isothermal amplification platforms, including LAMP and RPA, particularly when compared with highly sensitive laboratory-based molecular methods such as qPCR or nested PCR [30,38,39,40]. Genetic diversity within *C*Mhm represents another possible explanation for the discordant results. Multilocus sequence analyses have identified several genetically distinct *C*Mhm subgroups (A, B, and C) [8]. Such genetic diversity could potentially affect primer or probe binding if polymorphisms occur within assay-targeted regions; however, this possibility could not be directly evaluated in the present study. The two apparent false-positive results (samples Nos. 24 and 26), which were positive by the Pluslife^®^ FHM assay but negative by conventional PCR, also warrant consideration. One possible explanation is that these samples contained target DNA at concentrations detectable by the RHAM assay but below the detection limit of conventional gel-based PCR. However, because no conventional PCR amplicons were available for sequencing, the true infection status of these samples could not be independently resolved, and nonspecific assay signals or other technical factors cannot be excluded.

Furthermore, discrepancies between these two assays in detecting single and mixed infections warrant careful interpretation. The Pluslife^®^ FHM assay identified three cats with *C*Mhm–*Mhf* mixed infection that were classified as single-species infections by conventional PCR. One possible explanation is differential amplification efficiency when multiple target templates are present. In conventional PCR, differences in target abundance or amplification efficiency may reduce the detection of less abundant co-infecting organisms, although this mechanism was not directly investigated in the present study. Alternatively, the assay design of the Pluslife^®^ FHM platform may facilitate the simultaneous detection of multiple targets, potentially improving the identification of mixed infections. Similar advantages have been reported for RHAM-based molecular assays in previous validation studies, although additional analytical investigations are required to determine whether this mechanism contributes to the improved detection of mixed infections of feline hemotropic *Mycoplasma* species observed in the present study [19,20,21].

When these diagnostic findings are extrapolated to broader feline populations, including asymptomatic and shelter-housed cats, differences in infection prevalence and the magnitude and temporal dynamics of bacteremia should be considered. In asymptomatic carriers, hemoplasma bacteremia may occur at low levels and fluctuate over time, potentially approaching or falling below the detection limits of molecular assays [4,7]. Accordingly, the diagnostic sensitivity of the Pluslife^®^ FHM assay in asymptomatic populations may differ from the 93.22% observed in the clinically ill cats evaluated in the present study, particularly when bacterial loads are low. Furthermore, because predictive values depend on infection prevalence, the use of the assay in lower-prevalence screening populations would generally be expected to decrease the positive predictive value (PPV) and increase the negative predictive value (NPV), assuming similar sensitivity and specificity. Conversely, in shelter populations with a higher prevalence of hemoplasma infection, PPV would be expected to increase, potentially supporting the use of rapid testing for triage and informing appropriate infection-control or management decisions.

Despite its rapid turnaround time (~30 min) and closed-cartridge format, implementation of the RHAM platform in decentralized veterinary settings may face several practical challenges, particularly in resource-limited settings. First, instrument acquisition and recurring consumable costs may limit adoption by smaller veterinary practices, particularly when test costs are borne directly by clients. Although conventional PCR may achieve lower reagent costs when samples are processed in batches, meaningful economic comparisons should also account for laboratory infrastructure, personnel, sample transport, and turnaround time. Second, although the closed-cartridge format reduces the requirement for specialized molecular laboratory infrastructure, pre-analytical procedures—including accurate micropipetting, sample lysis, and cartridge loading—still require appropriate staff training to minimize handling errors, contamination, and invalid test results. Third, adherence to manufacturer-recommended transport and storage conditions is essential for maintaining reagent stability, particularly in tropical environments where high temperature and humidity may complicate product distribution and storage. Addressing these challenges will require context-appropriate pricing and distribution strategies, reliable supply and storage systems, and adequate staff training and quality-control procedures.

Several limitations of the present study should be acknowledged when interpreting these findings. First, the study population consisted exclusively of clinically affected cats recruited from a single veterinary teaching hospital, which may limit the generalizability of the findings to asymptomatic cats and populations from other clinical or geographic settings. Second, limited sample volumes prevented quantitative real-time PCR (qPCR) analysis and therefore precluded assessment of bacterial loads in false-negative samples. Consequently, the possibility that a low bacterial burden contributed to the observed diagnostic discordance could not be directly evaluated. Third, detailed hematological and clinical data—including anemia severity and regenerative status, packed cell volume, other blood cell indices, and febrile status—were not systematically available for all cats because specimens were obtained through a routine diagnostic submission workflow. The absence of these data prevented assessment of potential associations between species-specific infection, particularly *Mhf* and *C*Mhm, and anemia severity or other clinical manifestations. Finally, epidemiological and behavioral variables, including ectoparasite exposure or infestation, flea-control history, and indoor/outdoor lifestyle, were not systematically recorded for all enrolled cats. Consequently, the absence of these epidemiological data limited the assessment of potential risk factors or behavioral parameters that may facilitate transmission. Future multicenter studies involving larger and more diverse populations of clinically ill and asymptomatic cats, together with quantitative bacterial-load assessment and standardized collection of clinical and epidemiological data, are warranted to further validate the diagnostic performance of the Pluslife^®^ FHM assay and clarify its diagnostic utility across different feline populations.

## 5. Conclusions

The Pluslife^®^ FHM nucleic acid test card demonstrated strong diagnostic performance for the detection of feline hemotropic *Mycoplasma* infections, showing a sensitivity of 93.22%, specificity of 96.36%, overall accuracy of 94.74%, and precision (positive predictive value) of 96.49% relative to conventional PCR. Providing results in approximately 30 min, the assay provides rapid, species-specific molecular detection while reducing the requirement for specialized molecular laboratory infrastructure. Although six discordant results were observed among the 114 samples, the underlying causes could not be fully resolved due to the lack of bacterial load quantification and independent confirmation for all discordant specimens. Nevertheless, under the conditions evaluated in this study, the Pluslife^®^ FHM assay proved to be a reliable diagnostic tool when compared with conventional PCR. Its rapid turnaround time and simplified workflow support its potential use as a point-of-care molecular diagnostic aid for timely diagnosis and clinical management of feline hemoplasmosis, particularly in veterinary settings with limited access to centralized molecular laboratories.

## Figures and Tables

**Table 1:** Characteristics of 114 sick cats tested for hemotropic *Mycoplasma* infections at the Kasetsart University Veterinary Teaching Hospital during September 2025 to May 2026.

Characteristic		Cat (%)
Age	<1 year	9 (7.89)
	1–5 years	47 (41.23)
	6–10 years	41(35.96)
	>11 years	17 (14.91)
Gender	Male	76 (66.67)
	Female	38 (33.33)
Breed	Domestic Shorthair	87 (76.32)
	Scottish Fold	15 (13.16)
	Persian	6 (5.26)
	Exotic Shorthair	2 (1.75)
	American Shorthair	2 (1.75)
	Maine Coon	1 (0.88)
	Himalayan	1 (0.88)

**Table 2:** Diagnostic performance metrics and cross-tabulation of the Pluslife^®^ Feline Hemotropic *Mycoplasma* (FHM) nucleic acid test card compared with conventional PCR (*n* = 114).

Pluslife^®^ FHM	Conventional PCR		Diagnostic Performance Metric (%)
	Positive	Negative	
Positive	55	2	-
Negative	4	53	-
Diagnostic sensitivity (%)	-	-	93.22 (95% CI: 83.54–98.12)
Specificity (%)	-	-	96.36 (95% CI: 87.47–99.56)
Accuracy (%)	-	-	94.74 (95% CI: 88.90–98.05)
Precision (%)	-	-	96.49 (95% CI: 87.88–99.57)

**Table 3:** Nucleotide BLAST results of partial 16S ribosomal RNA gene sequences from the four false-negative cases identified by the Pluslife^®^ Feline Hemotropic *Mycoplasma* (FHM) nucleic acid test card compared with conventional PCR.

Sample ID	Accession Number	E-Value	Identity (%)	Species
66	PV584111.1	0.0	95.38	*C*Mhm
68	EU839985.1	0.0	99.32	*C*Mhm
105	EU839985.1	0.0	96.07	*C*Mhm
107	KC331022.1	0.0	99.49	*C*Mhm

**Table 4:** Comparison of the detection frequency and proportion of hemotropic *Mycoplasma* infections in 114 sick cats presented at Kasetsart University Veterinary Teaching Hospital between September 2025 and May 2026 using the Pluslife^®^ Feline Hemotropic *Mycoplasma* (FHM) nucleic acid test card versus conventional PCR.

Hemotropic *Mycoplasma* Species	Pluslife^®^ FHM [No. (%)]	Conventional PCR [No. (%)]
*C*Mhm	48 (42.11)	52 (45.61)
*Mhf*	5 (4.39)	6 (5.26)
*C*Mt	1 (0.88)	1 (0.88)
*C*Mhm + *Mhf*	3 (2.63)	0 (0.00)

*C*Mhm: ‘*Candidatus* Mycoplasma haemominutum’; *Mhf*: *Mycoplasma haemofelis*; *C*Mt: ‘*Candidatus* Mycoplasma turicensis’.

## Data Availability

The original contributions presented in this study are included in the article/Supplementary Material. Further inquiries can be directed to the corresponding author.

## Supplementary Materials

The following supporting information can be downloaded at: https://www.mdpi.com/article/10.3390/vetsci13090978/s1, Table S1: Individual genomic detection of ‘*Candidatus* Mycoplasma haemominutum’ (*C*Mhm), *Mycoplasma haemofelis* (*Mhf*), and ‘*Candidatus* Mycoplasma turicensis’ (*C*Mt) in 114 sick cats using the Pluslife^®^ Feline Hemotropic *Mycoplasma* (FHM) nucleic acid test card compared with conventional PCR and DNA sequencing.
