# Supplementary material for: Molecular Detection of Feline Hemotropic Mycoplasma from Blood Samples of Sick Cats: A Comparative Study Between a Commercial Nucleic Acid Test Card and Conventional Polymerase Chain Reaction
Source: Vet Sci. 2026 Sep 17;13(9):978. doi: 10.3390/vetsci13090978 (PMC13612060; doi:10.3390/vetsci13090978)
Supplement: Supplementary file 1 [file vetsci-13-00978-s001.zip › vetsci-4538446-supplementary.pdf]

Table S1. Individual genomic detection of '*Candidatus* Mycoplasma haemominutum' (CMhm), *Mycoplasma haemofelis* (*Mhf*), and '*Candidatus* Mycoplasma turicensis' (*C*Mt) in 114 sick cats using the Pluslife® Feline Hemotropic *Mycoplasma* (FHM) nucleic acid test card compared with conventional PCR and DNA sequencing.

No	Pluslife® FHM			PCR	No	Pluslife® FHM			PCR
	***C*Mhm**	***Mhf***	***C*Mt**	**(DNA sequencing)**		***C*Mhm**	***Mhf***	***C*Mt**	**(DNA sequencing)**
1	negative	negative	negative	negative	58	positive	positive	negative	positive (CMhm)
2	negative	negative	negative	negative	59	positive	negative	negative	positive (CMhm)
3	negative	negative	negative	negative	60	positive	negative	negative	positive (CMhm)
4	negative	negative	negative	negative	61	negative	negative	negative	negative
5	negative	negative	negative	negative	62	negative	negative	negative	negative
6	negative	negative	negative	negative	63	positive	negative	negative	positive (CMhm)
7	negative	negative	negative	negative	64	positive	negative	negative	positive (CMhm)
8	negative	negative	negative	negative	65	negative	negative	negative	negative
9	negative	negative	negative	negative	66	negative	negative	negative	positive (CMhm)
10	negative	negative	negative	negative	67	negative	negative	negative	negative
11	negative	negative	negative	negative	68	negative	negative	negative	positive (CMhm)
12	negative	negative	negative	negative	69	positive	negative	negative	positive (CMhm)
13	negative	negative	negative	negative	70	positive	negative	negative	positive (CMhm)
14	negative	negative	negative	negative	71	positive	negative	negative	positive (CMhm)
15	negative	negative	negative	negative	72	positive	negative	negative	positive (CMhm)
16	negative	negative	negative	negative	73	positive	negative	negative	positive (CMhm)
17	negative	negative	negative	negative	74	positive	negative	negative	positive (CMhm)
18	negative	negative	negative	negative	75	positive	negative	negative	positive (CMhm)
19	negative	negative	negative	negative	76	positive	negative	negative	positive (CMhm)
20	negative	negative	negative	negative	77	positive	negative	negative	positive (CMhm)
21	negative	negative	negative	negative	78	positive	negative	negative	positive (CMhm)
22	negative	negative	negative	negative	79	positive	negative	negative	positive (CMhm)
23	negative	negative	negative	negative	80	negative	positive	negative	positive (*Mhf*)
24	positive	negative	negative	negative	81	positive	negative	negative	positive (CMhm)
25	negative	negative	negative	negative	82	positive	negative	negative	positive (CMhm)
26	positive	negative	negative	negative	83	negative	positive	negative	positive (*Mhf*)
27	negative	negative	negative	negative	84	positive	negative	negative	positive (CMhm)
28	negative	negative	negative	negative	85	positive	negative	negative	positive (CMhm)
29	negative	negative	negative	negative	86	positive	negative	negative	positive (CMhm)
30	negative	negative	negative	negative	87	positive	negative	negative	positive (CMhm)
31	negative	negative	negative	negative	88	positive	negative	negative	positive (CMhm)
32	negative	negative	negative	negative	89	positive	negative	negative	positive (CMhm)
33	negative	negative	negative	negative	90	positive	negative	negative	positive (CMhm)
34	negative	negative	negative	negative	91	positive	negative	negative	positive (CMhm)
35	negative	negative	negative	negative	92	negative	negative	positive	positive (CMt)
36	negative	negative	negative	negative	93	positive	negative	negative	positive (CMhm)
37	negative	negative	negative	negative	94	positive	negative	negative	positive (CMhm)
38	negative	negative	negative	negative	95	positive	negative	negative	positive (CMhm)
39	negative	negative	negative	negative	96	negative	positive	negative	positive (*Mhf*)
40	negative	negative	negative	negative	97	positive	negative	negative	positive (CMhm)
41	negative	negative	negative	negative	98	positive	negative	negative	positive (CMhm)
42	negative	negative	negative	negative	99	positive	negative	negative	positive (CMhm)
43	negative	negative	negative	negative	100	positive	negative	negative	positive (CMhm)
44	negative	negative	negative	negative	101	positive	negative	negative	positive (CMhm)
45	negative	negative	negative	negative	102	positive	negative	negative	positive (CMhm)
46	negative	negative	negative	negative	103	positive	negative	negative	positive (CMhm)
47	negative	negative	negative	negative	104	positive	negative	negative	positive (CMhm)
48	negative	negative	negative	negative	105	negative	negative	negative	positive (CMhm)
49	negative	negative	negative	negative	106	positive	negative	negative	positive (CMhm)
50	negative	negative	negative	negative	107	negative	negative	negative	positive (CMhm)
51	negative	negative	negative	negative	108	positive	negative	negative	positive (CMhm)
52	positive	positive	negative	positive (*Mhf*)	109	positive	negative	negative	positive (CMhm)
53	positive	negative	negative	positive (CMhm)	110	positive	negative	negative	positive (CMhm)
54	positive	negative	negative	positive (CMhm)	111	positive	negative	negative	positive (CMhm)
55	positive	negative	negative	positive (CMhm)	112	positive	negative	negative	positive (CMhm)
56	negative	positive	negative	positive (*Mhf*)	113	positive	negative	negative	positive (CMhm)
57	negative	positive	negative	positive (*Mhf*)	114	positive	positive	negative	positive (CMhm)

positive: detected nucleic acid; negative: undetected nucleic acid
